# How Often Do Large Language Models Agree with Each Other—And with the Truth? A Consensus- and Complexity-Stratified Analysis of Data Extraction for Neuroimaging AI

**DOI:** 10.3390/jcm15156005

**Published:** 2026-08-02

**Authors:** Nafiye Sanlier, Umid Sulaimanov, Ariorad Moniri, Behman Demir, Gular Ismayilova, Melih Yucel Sanlier, Ugur Erginoglu, Ahmed Rasim Bayramoglu, Maryam Sabah Al-Jebur, Simon Gashaw Ammanuel, Erkin Otles, Abdullah Keles, Ufuk Erginoglu, Mustafa K. Baskaya

**Affiliations:** 1 Department of Neurological Surgery, School of Medicine and Public Health, University of Wisconsin-Madison, Madison, WI 53792, USA; 2 School of Medicine, Acibadem University, Istanbul 34752, Turkey; 3 Department of Neurosurgery, University of Health Sciences, Istanbul Training and Research Hospital, Istanbul 34098, Turkey; 4 Department of Emergency Medicine, Istanbul Medeniyet University, Prof. Dr. Suleyman Yalcin City Hospital, Istanbul 34722, Turkey; 5 Department of Otolaryngology-Head and Neck Surgery, Basaksehir State Hospital, Istanbul 34480, Turkey; 6 BerbeeWalsh Department of Emergency Medicine, School of Medicine and Public Health, University of Wisconsin-Madison, Madison, WI 53792, USA

**Keywords:** artificial intelligence, neuroimaging, data extraction, evidence synthesis, large language models, variable complexity

## Abstract

**Background:** The reliable integration of large language models (LLMs) into neuroimaging data extraction workflows remains unresolved. Prior benchmarking shows that exact-match accuracy underestimates LLM extraction performance, but whether inter-model consensus and variable complexity can guide automation remains unclear. We evaluated whether inter-model consensus can serve as a confidence signal for human–artificial intelligence (AI) extraction and can guide complexity-stratified workflow triage. **Methods:** Four frontier LLMs were queried via OpenRouter with an identical zero-shot structured prompt to extract 22 predefined variables from 91 peer-reviewed neuroimaging AI articles, yielding 2002 article–variable items per model. Variables were stratified a priori into low- (*n* = 7), medium- (*n* = 8), and high-complexity (*n* = 7). Performance was compared with an expert reference using exact-match and semantic-equivalence accuracy. Item-level consensus and five triage strategies characterized the efficiency–accuracy trade-off. **Results:** Semantic-equivalence accuracy converged to 80.5–83.4% across models despite approximately ten percentage-point exact-match differences. Unanimous 4/4 consensus occurred in 45.6% (910/1994) of items, with exact-match accuracy of 85.8%, rising to 95.3% after semantic normalization; however, 14.2% still failed to match the reference. Reliability was complexity-dependent: 96.6% for low-complexity variables, 73.2% for medium-complexity variables, and 38.1% for high-complexity variables. A hybrid strategy auto-accepting 4/4 items and routing 3/4 items to rapid verification reduced estimated review effort by approximately 59%. **Conclusions:** Inter-model consensus is useful, but it is incomplete and depends on variable complexity. We show that LLM-assisted extraction in neuroimaging AI is a complexity-stratified workflow design problem: low-complexity neuroimaging variables may be selectively automated, while medium-complexity variables require rapid verification, and high-complexity methodological variables should remain human-led.

## 1. Introduction

The biomedical literature is expanding faster than it can be reliably synthesized. In the conduct of systematic reviews and meta-analyses, converting diverse primary reports into harmonized evidence tables is one of the most effort-intensive and failure-prone stages, since it must be carried out consistently across many reviewers and studies. A reproducibility study of 201 systematic reviews of adverse events showed that 66.8% of meta-analyses and 85.1% of reviews contained at least one data-extraction error, indicating that such errors are common and can materially affect downstream synthesis [1,2]. Earlier natural language processing pipelines achieved partial automation but depended on task-specific training data and narrow extraction schemas that limited generalizability across review contexts [3].

Large language models (LLMs) offer a more adaptable alternative because they can extract structured information from unstructured text in a zero-shot setting without task-specific retraining [4,5]. Early evidence suggests that LLM-assisted workflows can improve efficiency and support semi-automated extraction, but performance is variable across tasks and tends to decline as variables become more interpretive or methodologically complex [2,6,7]. This challenge is particularly relevant for artificial intelligence (AI) in neuroimaging—predominantly magnetic resonance imaging (MRI)- and computed tomography (CT)-based models [8]—where reporting remains heterogeneous and many target variables require synthesis across multiple sections of an article rather than direct, sentence-level retrieval. Although reporting frameworks such as the Checklist for Artificial Intelligence in Medical Imaging (CLAIM) and the Transparent Reporting of a multivariable prediction model for Individual Prognosis Or Diagnosis plus Artificial Intelligence (TRIPOD + AI) define the information that should be reported, adherence remains inconsistent; therefore, extraction often still requires contextual interpretation even when variables are nominally well defined [9,10]. Recent methodological commentary has thus argued that an evaluation of LLMs for evidence synthesis should extend beyond raw accuracy to workflow-relevant dimensions such as error tolerance, reference-standard alignment, and implementation safeguards [4,11].

Two previously published studies have addressed this domain using the same 91-article corpus and 22-variable extraction schema applied herein. The first was a systematic methodological review of neuroimaging AI studies, which established the corpus, a predefined extraction schema comprising selected methodological and reporting variables informed by CLAIM/TRIPOD + AI, and the expert consensus reference standard [8]. The schema was not intended to provide a comprehensive assessment of adherence to either reporting guideline or to encompass every domain relevant to clinical generalizability, equity, and population representativeness. The second was a multi-model benchmark in which the same four frontier LLMs were evaluated on the corpus under strict exact-match accuracy against the reference standard [12]. That benchmark established baseline extraction performance and identified a striking representational mismatch effect: all four models scored 0% exact-match accuracy on the main performance metric variable despite correctly recovering the reported metric in approximately 90% of articles under semantic review, indicating that exact-match scoring systematically underestimates true semantic performance. However, the benchmark was designed as a model-comparison study and left several workflow-critical questions open: (1) it did not evaluate inter-model consensus as an operational confidence signal; (2) it did not apply prespecified semantic-equivalence adjudication beyond a single illustrative variable; and (3) it did not stratify the reliability of consensus across variable complexity tiers as a basis for triage. The present study is a companion workflow-oriented secondary analysis designed to address these gaps directly. The target variables, reference labels, and LLM outputs were predefined; therefore, this secondary analysis evaluated the existing schema rather than expanding it. Each of these is a workflow design question that determines when outputs may be auto-accepted, when they require rapid verification, and when they must be reserved for full expert review.

To address these questions, we evaluated four contemporary frontier LLMs—Google Gemini 3 Pro Preview, Anthropic Claude Opus 4.5, Perplexity Sonar Pro, and OpenAI GPT 5.2—on the same 91-article corpus and predefined 22-variable schema used in the prior benchmark [8,12]. All models were evaluated using the same fixed zero-shot prompt and standardized article representation. Rather than focusing solely on raw extraction accuracy, we examined: (i) semantic near-miss matching as a complement to exact-match scoring; (ii) consensus-stratified accuracy across 1/4 to 4/4 agreement levels; (iii) complexity-dependent reliability of consensus; and (iv) the empirical efficiency–accuracy frontier under prespecified triage strategies. Our aim was to determine whether inter-model consensus can serve as a practical confidence signal for human–AI hybrid extraction, and how semantic normalization and variable complexity affect its operational reliability within this controlled baseline, rather than to define the maximum performance achievable through task-specific optimization.

## 2. Materials and Methods

### 2.1. Study Design and Extraction Pipeline

Using the same 91-article corpus as our companion benchmark [12], this study performs a workflow-focused secondary analysis, reusing that benchmark’s structured LLM outputs and its curated reference dataset rather than generating new extractions. The corpus, 22 predefined target variables, extraction schema, optical character recognition (OCR) preprocessing, LLM querying procedure, prompt, and expert reference standard were identical to those described in detail in the companion benchmark. The extraction targets and reference annotations were fixed before the present consensus- and complexity-stratified analyses began, and no new extraction domains were introduced in this secondary analysis. Here we only summarize the elements essential to the present analyses.

The corpus comprised the same 91 peer-reviewed primary studies that met the eligibility criteria and were included in the qualitative synthesis of the previously published systematic methodological review, registered in the International Prospective Register of Systematic Reviews (PROSPERO CRD420261284068) [8]. References cited in that review for background, methodological, and reporting-guideline purposes were not part of the extraction corpus. The complete list of the 91 analyzed studies is provided in Supplementary Table S3. All included articles were born-digital, publisher-issued portable document format (PDF) files with embedded text; no scanned or image-only files were included. To standardize input across models, Mistral OCR performed layout-aware PDF-to-text conversion rather than model-specific native parsing. Outputs underwent automated checks of completeness, expected sections, and OCR artifacts, followed by manual spot-checks by two reviewers focusing on the Methods, Results, performance tables, numerical values, and reported performance metrics. No systematic failures were identified, and no article was excluded. Four frontier LLMs were examined: (1) Google Gemini 3 Pro Preview, (2) Anthropic Claude Opus 4.5, (3) Perplexity Sonar Pro, and (4) OpenAI GPT 5.2. All four LLMs were queried via OpenRouter on 2 February 2026 using an identical zero-shot structured JavaScript Object Notation (JSON) prompt at a fixed temperature of 0.3. Full model snapshot identifiers, sampling parameters, and the verbatim prompt are reported in the companion benchmark and its Supplementary Data S1 [12]. No few-shot examples, evidence retrieval, stepwise extraction, or alternative context-selection strategy were used; all models received the same standardized article representation.

The predefined 22-variable extraction schema, comprising selected methodological and reporting fields informed by CLAIM and TRIPOD + AI [8,9,10], was stratified *a priori* into low- (*n* = 7), medium- (*n* = 8), and high-complexity (*n* = 7) tiers according to the nature of the extraction task. It was not designed as a comprehensive checklist of adherence to either reporting guideline. The full variable lists by tier are provided in Supplementary Table S1. Each article × variable combination yielded one item, giving 91 × 22 = 2002 item-level evaluations per model.

**Figure 1:**
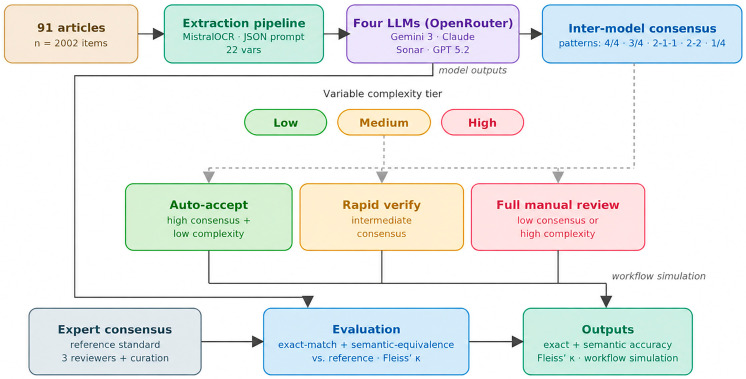
**Complexity-stratified large language model data extraction and workflow framework.** Structured extraction of 22 prespecified variables from 91 neuroimaging artificial intelligence (AI) articles was performed using four frontier large language models (LLMs) accessed via OpenRouter under identical sampling parameters. Inter-model consensus and a priori variable complexity were jointly used to define triage strategies for automatic acceptance, rapid human verification, and full manual review. Outputs were evaluated against a curated expert consensus reference standard across 2002 article–variable items per model. Created in BioRender. Sanlier, N. (2026) https://BioRender.com/e3bozlc.

The gold-standard annotations originated from the consensus dataset built for this corpus in the prior methodological review [8], where three reviewers applied a CLAIM/TRIPOD + AI-aligned form. For the present work, this dataset underwent a further verification round: authors NS and US re-coded every variable–article pair against the primary PDFs independently, with author BD resolving any remaining discrepancies. The same refined reference standard was used in the companion benchmark [12] and is used here. The overall data extraction and evaluation workflow is summarized in Figure 1.

### 2.2. Semantic Near-Miss Adjudication

Because exact-match scoring can misclassify semantically equivalent responses as incorrect when they only differ from the reference standard in surface form, a prespecified semantic near-miss adjudication procedure was applied as a secondary evaluation. While the companion benchmark applied a single illustrative semantic check to the main performance metric variable only [12], the present study extends that approach by applying a uniform semantic rule set across all 22 variables and all four LLMs. Responses were credited as semantically correct when it was judged to be meaning-equivalent to the expert reference despite minor differences that were confined to (i) abbreviation versus expanded form, (ii) capitalization, (iii) synonymous lexical variants, or (iv) minor wording differences that did not alter categorical meaning. Responses were not credited as semantic matches when wording changes altered categorical assignment, methodological interpretation, or level of evidentiary support. Three reviewers (authors MYS, ARB, GI) independently performed the semantic adjudication using a written rule set developed and finalized before adjudication began, with any disagreements resolved by consensus.

### 2.3. Consensus Definition

Inter-model consensus was evaluated at the item level. For each article–variable pair, the four normalized outputs were compared, and the agreement pattern was recorded as 4/4 (all four identical), 3/4 (three identical, one divergent), 2-1-1 (one identical pair with the remaining two distinct from each other), 2-2 (two distinct identical pairs), or 1/4 (all four distinct). Two model outputs were treated as identical when they matched after the same canonical normalization used for ground-truth matching in the companion benchmark (e.g., expansion of standard abbreviations such as “DL” to “deep learning” and removal of numeric formatting characters such that “*n* = 1234” and “1234” were treated as equivalent), so that responses differing only in surface form were counted as agreeing [12]. Patterns with a unique plurality output (4/4, 3/4, and 2-1-1) formed the primary consensus gradient; tied 2-2 and fully discordant 1/4 patterns were treated as non-actionable-consensus strata and analyzed separately by candidate-set reference alignment. For consensus-stratified analyses, items in which all four models returned “not reported” were excluded because they did not yield an informative consensus signal.

### 2.4. Statistical Analysis

The primary outcome was exact-match accuracy of the inter-model plurality answer in actionable strata (4/4, 3/4, and 2-1-1). The secondary outcome was semantic-equivalence accuracy. For strata without an actionable consensus (2-2, 1/4), candidate-set reference alignment was reported as the proportion of items in which the expert reference matched at least one model output exactly or semantically, reflecting recoverability under full human review. All proportions were reported with Wilson score 95% confidence intervals without continuity correction. Inter-rater agreement was summarized using Fleiss’ κ in two configurations: inter-model agreement among the four LLMs, and system-level agreement that additionally included the expert reference as a fifth rater. κ values were interpreted using Landis and Koch benchmarks: <0.00 (poor), 0.00–0.20 (slight), 0.21–0.40 (fair), 0.41–0.60 (moderate), 0.61–0.80 (substantial), and 0.81–1.00 (almost perfect). Model-level extraction accuracy, omission and misclassification rates, and model-to-ground-truth Cohen’s κ values for this corpus are reported in the companion benchmark and are not repeated here [12].

Workflow-oriented analyses were prespecified across four dimensions: (i) semantic near-miss analysis comparing exact-match and semantic-equivalence performance at the consensus-stratum level; (ii) consensus-stratified exact and semantic accuracy for actionable plurality strata (4/4, 3/4, 2-1-1), and candidate-set alignment for no-consensus strata (2-2, 1/4); (iii) complexity-stratified analyses evaluating whether consensus carried different implications across low-, medium-, and high-complexity variables; and (iv) workflow simulation modeling five prespecified triage strategies. Reviewer workload was modeled using a prespecified weighted-effort framework with three decision categories assigned relative effort weights: automatic acceptance = 0, rapid human verification = 0.20, and full manual review = 1.00. Relative workload reduction for each strategy was estimated against a baseline in which all items underwent full manual review under this weighting scheme. Because the same articles and variables were evaluated across all four models, agreement and workflow analyses respected the repeated-measures structure of the data. Consistent with the workflow design focus of the study, descriptive and reliability-oriented analyses were prioritized over omnibus model-comparison testing. Statistical significance was defined as *p* < 0.05.

## 3. Results

### 3.1. Exact-Match Accuracy and Inter-Model Consensus

**Figure 2:**
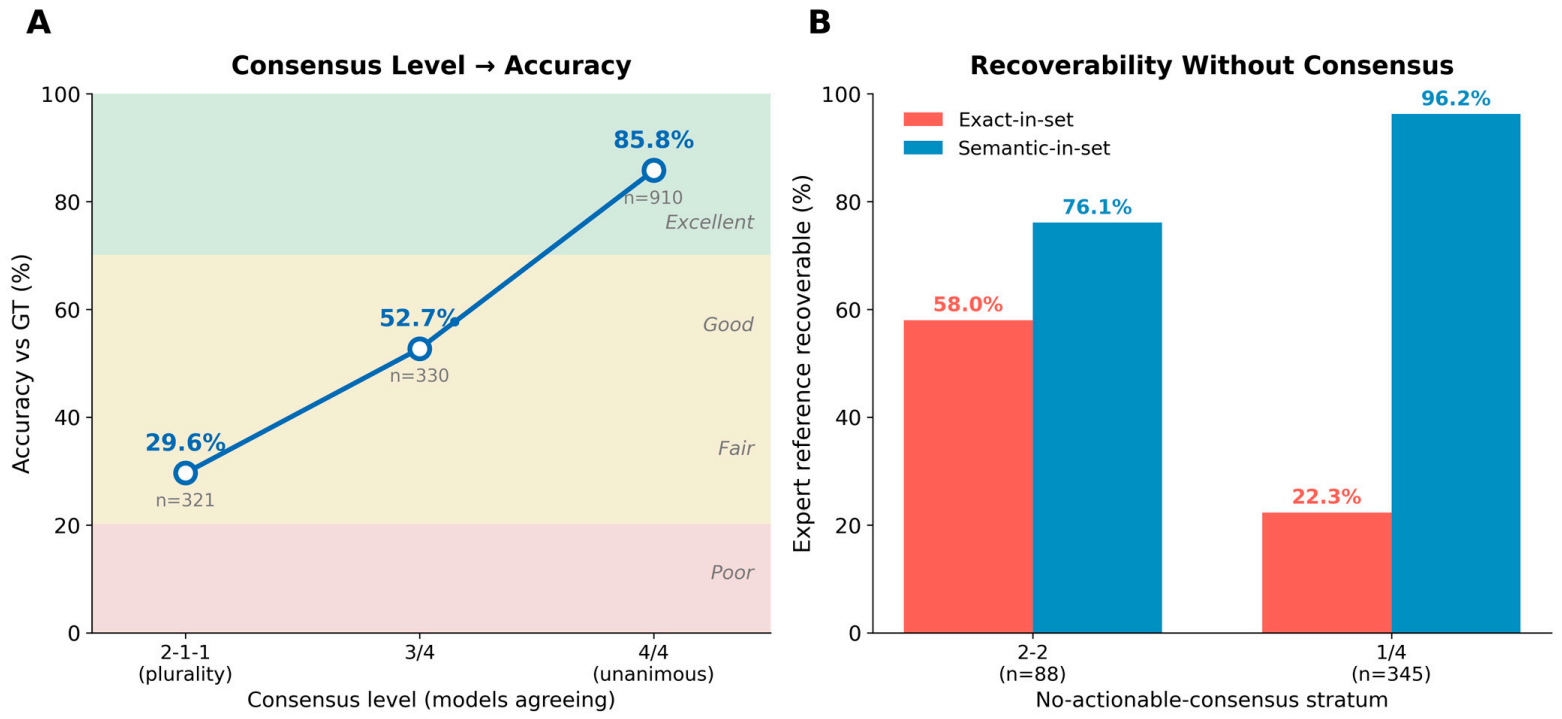
**Consensus accuracy and recoverability.** (**A**) Exact-match accuracy of the consensus (plurality) answer across the three actionable-consensus strata (2-1-1, 3/4, 4/4; n shown), increasing monotonically with agreement. (**B**) Candidate-set reference alignment (exact-in-set, semantic-in-set) for the no-consensus strata (2-2, 1/4); the reference was semantically recoverable in 96.2% of 1/4 items.

Across the 1994 items (eight items were excluded because all four models returned “not reported”), exact-match accuracy of the consensus answer increased monotonically across the three actionable-consensus strata. Unanimous 4/4 agreement yielded the highest exact-match accuracy at 85.8% (781/910), with progressively lower accuracy at 3/4 (52.7%, 174/330) and 2-1-1 plurality agreement (29.6%, 95/321). The 2-2 (*n* = 88) and 1/4 (*n* = 345) patterns yielded no unique plurality answer and were characterized separately (Section 3.2). Despite this clear gradient, unanimity was not equivalent to correctness: 14.2% of unanimously agreed items (129/910) still failed to match the expert reference standard. The relationship between consensus level and ground-truth accuracy is shown in Figure 2. 

### 3.2. Impact of Semantic Matching on Accuracy Estimates

Exact-match evaluation systematically underestimated performance when model outputs were meaning-equivalent but lexically non-identical. As reported in the companion benchmark, exact-match accuracy across the four models ranged from 46.5% to 56.4% under strict scoring [12]. Under prespecified semantic normalization applied uniformly across all 22 variables, the four models converged to a narrow 80.5–83.4% accuracy range (Table 1), with per-model gains of approximately 26–34 percentage points and inter-model spread compressed from approximately ten percentage points (exact-match) to under three (semantic).

**Figure 3:**
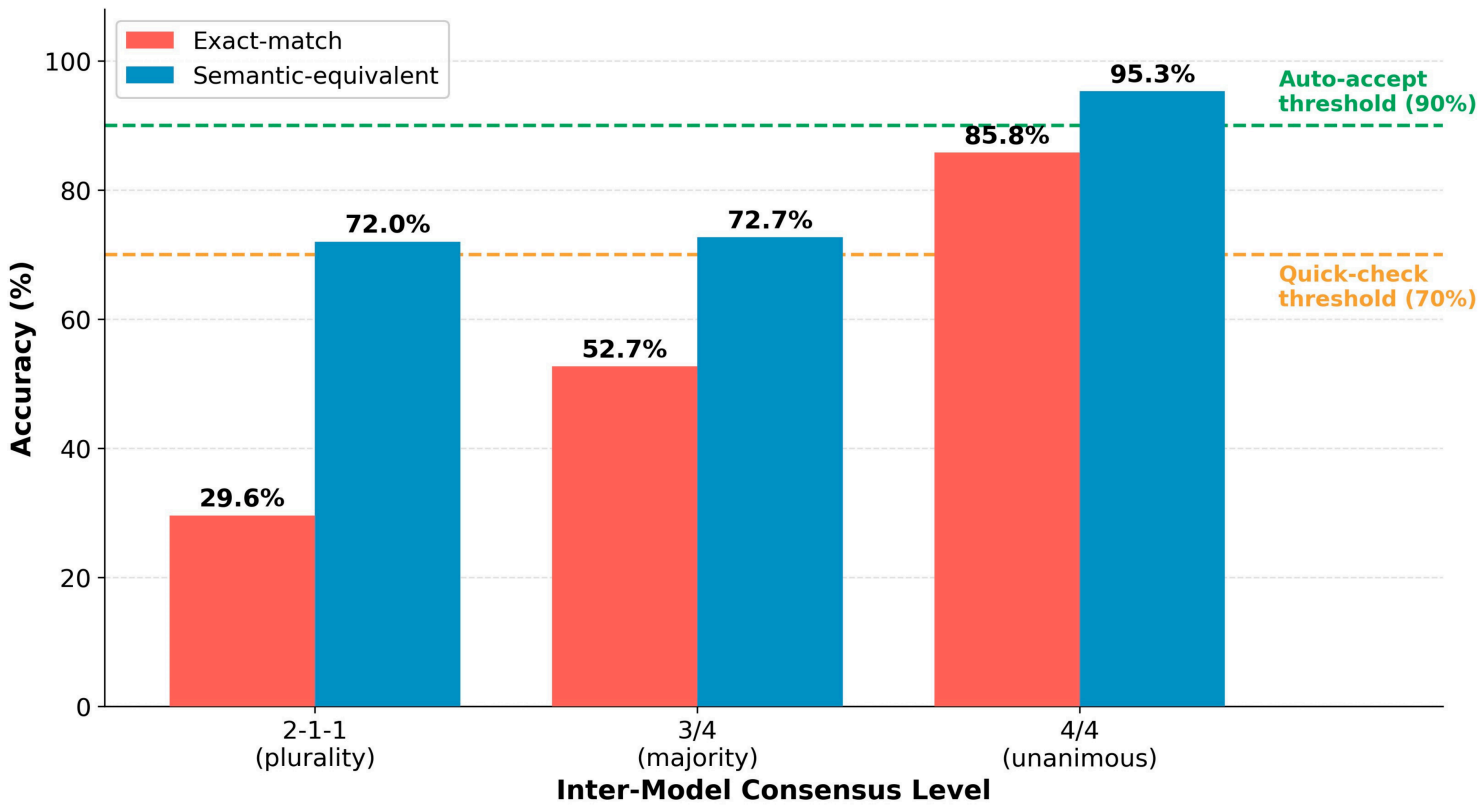
**Impact of semantic matching on the consensus–accuracy relationship.** Exact-match versus semantic-equivalence accuracy of the consensus (plurality) answer at each actionable-consensus stratum (2-1-1, 3/4, 4/4). Semantic normalization raised accuracy at every stratum, most steeply at 2-1-1. Dashed lines mark the operational accuracy thresholds used in the workflow simulation.

Within the unanimous-agreement stratum (*n* = 910), incorrect items decreased from 129 to 43 under semantic normalization, increasing 4/4 consensus accuracy from 85.8% to 95.3% (867/910). Relative gains were largest at lower consensus levels: accuracy rose from 52.7% to 72.7% at 3/4, and from 29.6% to 72.0% at 2-1-1 plurality. The effect of semantic normalization on the consensus–accuracy relationship is shown in Figure 3. Gains were most pronounced for variables admitting multiple permissible lexical forms, and smallest for high-complexity methodological variables. For strata without actionable consensus, candidate-set reference alignment was assessed as whether the expert reference appeared among the four model outputs. In 2-2 items (*n* = 88), exact and semantic alignment were 58.0% (51/88) and 76.1% (67/88), respectively. In 1/4 items (*n* = 345), exact alignment was low at 22.3% (77/345), but semantic alignment reached 96.2% (332/345).

### 3.3. Consensus Level, Variable Complexity, and Triage Implications

**Figure 4:**
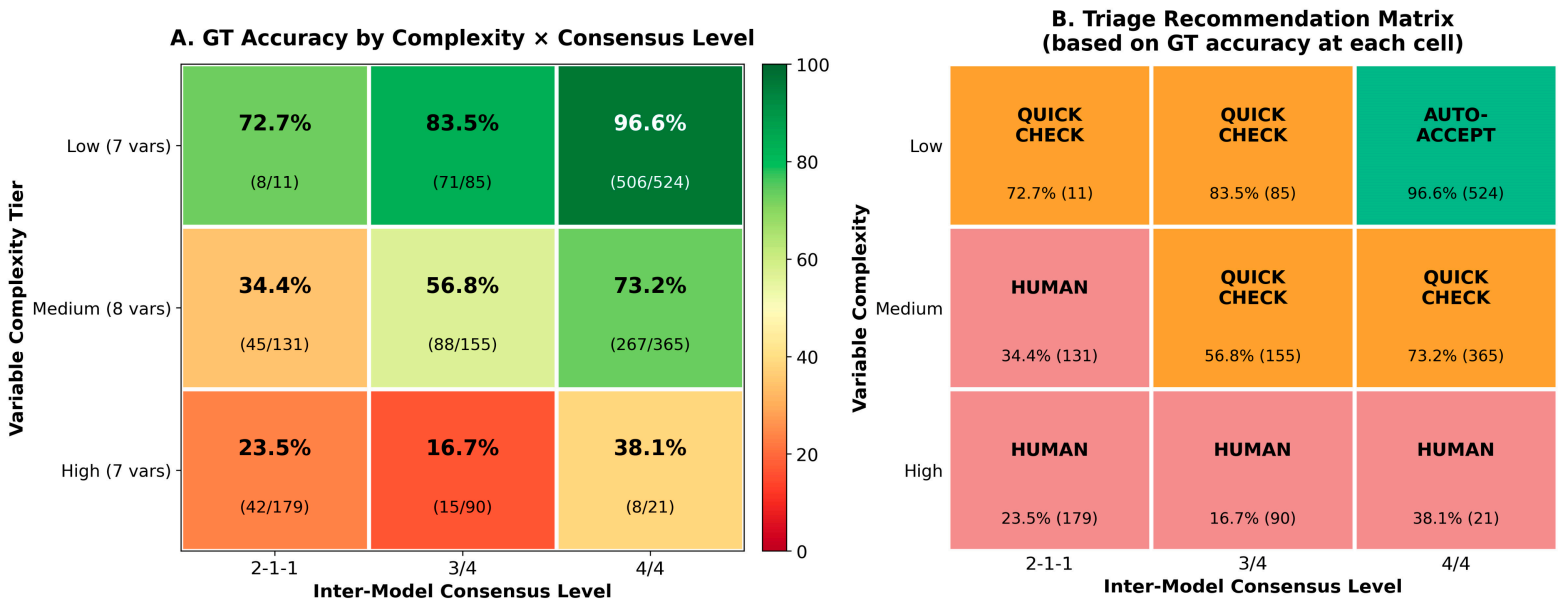
**Consensus–complexity accuracy and derived triage framework.** (**A**) Exact-match accuracy of the consensus (plurality) answer across the three actionable-consensus strata (2-1-1, 3/4, 4/4) by variable complexity; cells show accuracy and (correct/total). (**B**) Triage action per cell derived from (**A**): automatic acceptance (≥90%), rapid verification (50–89%), or full manual review (<50%). Consensus is reliable for low-complexity variables but insufficient for high-complexity variables at every agreement level. (GT: Ground Truth).

The relationship between consensus and correctness varied substantially across the prespecified complexity tiers. Using the 7-8-7 stratification defined at the full dataset level (*n* = 2002), variables were partitioned into 637 low-complexity, 728 medium-complexity, and 637 high-complexity items, with consensus-stratified analyses conducted within the 1994 items (Figure 4A). For low-complexity variables, consensus was highly informative: exact-match accuracy reached 96.6% (506/524) at 4/4 agreement, 83.5% (71/85) at 3/4, and 72.7% (8/11) at 2-1-1 plurality. For medium-complexity variables, accuracy rose with consensus but plateaued at a lower ceiling: 73.2% (267/365) at 4/4, 56.8% (88/155) at 3/4, and 34.4% (45/131) at 2-1-1. High-complexity variables showed the weakest relationship between consensus and correctness: 38.1% (8/21) at 4/4, 16.7% (15/90) at 3/4, and 23.5% (42/179) at 2-1-1 agreement. These results demonstrate a clear interaction between consensus level and variable complexity. The resulting consensus–complexity accuracy matrix is summarized as an operational triage framework. The corresponding framework is shown in Figure 4B.

### 3.4. Inter-Model Agreement and Correlated Error Patterns

**Figure 5:**
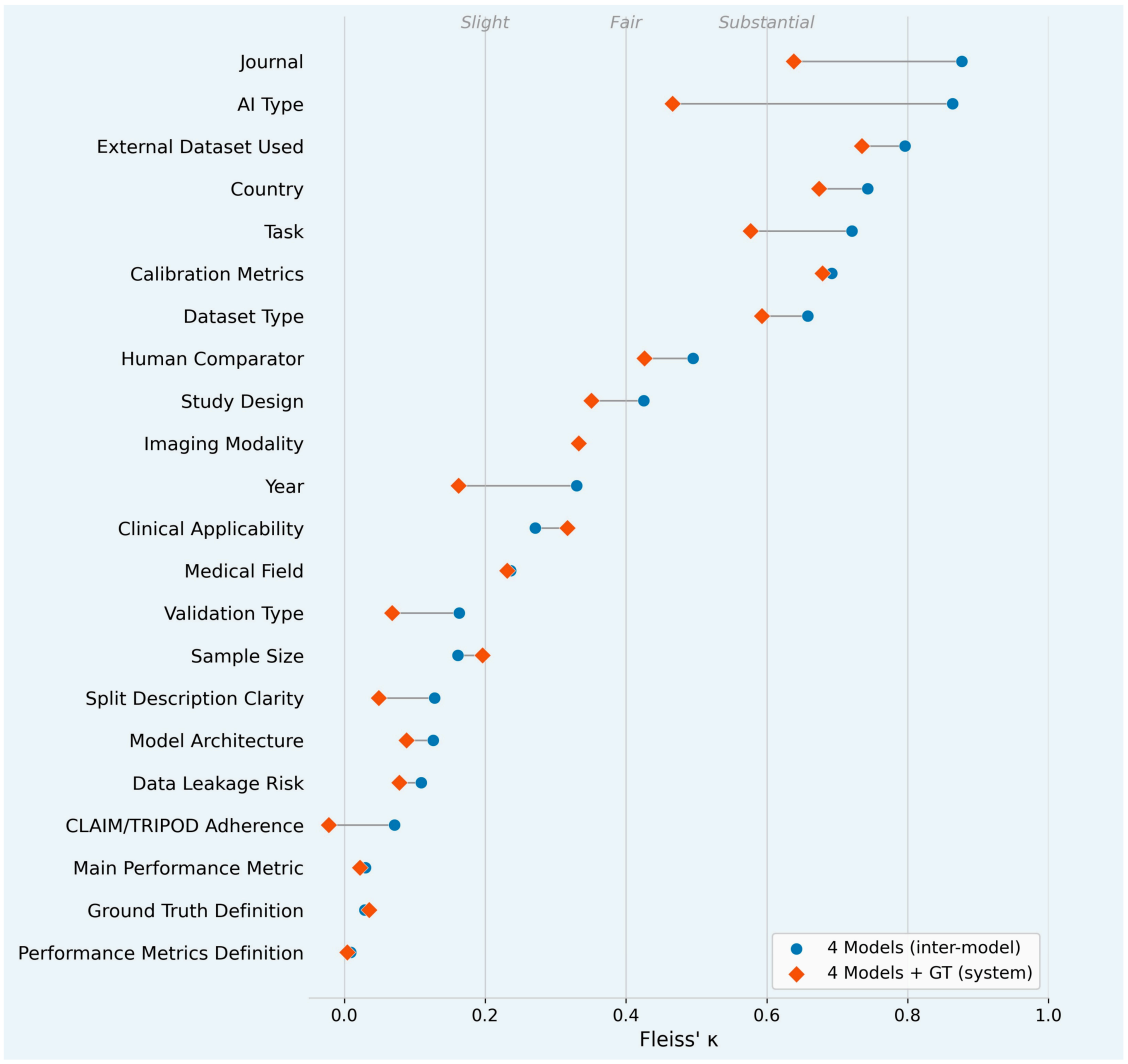
**Fleiss’κ: inter-model versus system-level agreement.** Variable-level Fleiss’κ under two configurations: inter-model agreement among the four large language models (LLMs) (blue circles) and system-level agreement including the expert ground truth as a fifth rater (orange diamonds). Vertical reference lines indicate Landis and Koch interpretation thresholds (slight, fair, substantial). Variables are ranked by descending inter-model κ. Mean inter-model κ = 0.376, and mean system-level κ = 0.304. *n* = 91 articles, 22 variables.

Multi-rater Fleiss’ κ was computed in two configurations to test whether inter-model agreement reflects independent validation or correlated error tendencies. Variable-level Fleiss’ κ among the four LLMs alone was 0.376, and when the expert reference was added as a fifth rater, system-level Fleiss’ κ fell to 0.304. This drop indicates that consensus among the models is systematically higher than agreement between the models and the reference standard, consistent with shared rather than independent error tendencies. The pattern was most pronounced for categorical variables with limited canonical forms: AI type, for example, reached κ = 0.864 across the four models but dropped sharply on the addition of the reference because the ground-truth encoding used a different canonical label format. Other variables in this pattern included journal (inter-model κ = 0.877), whereas the CLAIM/TRIPOD adherence, ground-truth definition, validation type, and main performance metric showed consistently low κ values regardless of configuration (Figure 5).

Pairwise model-to-model Cohen’s κ values ranged from 0.526 to 0.582, consistently exceeding the corresponding model-to-ground-truth values reported in the companion benchmark [12]. This pattern parallels the Fleiss’ κ comparison, with inter-model agreement consistently exceeding model-to-reference agreement.

### 3.5. Workflow Simulation and Efficiency–Accuracy Trade-Off

To evaluate the practical impact of consensus-based extraction, five prespecified triage strategies were simulated across the 1994 items. In the baseline scenario, all items underwent full human review (100% manual workload). Automatic acceptance of unanimous 4/4 items alone automated 910/1994 items (45.6%), but the exact-match error rate among auto-accepted items remained 14.2%, and the semantic error rate was 4.7% after normalization.

By lowering the threshold to ≥3/4, the consensus increased automation to 1240/1994 items (62.2%) but raised the auto-accept exact-match error rate to 23.0%. A hybrid strategy auto-accepting 4/4 items and routing 3/4 items to rapid human verification reduced estimated effective reviewer workload by approximately 59% while preserving the same auto-accept error profile as the unanimous-only subset. This estimate was robust to the assumed quick-check effort weight, remaining above 50% across plausible values (0.10–0.50; Supplementary Table S2). A conservative complexity-restricted strategy auto-accepting only low-complexity items with 4/4 consensus automated 524/1994 items (26.3%) and reduced the exact-match error rate among automatically processed items to 3.4%.

**Figure 6:**
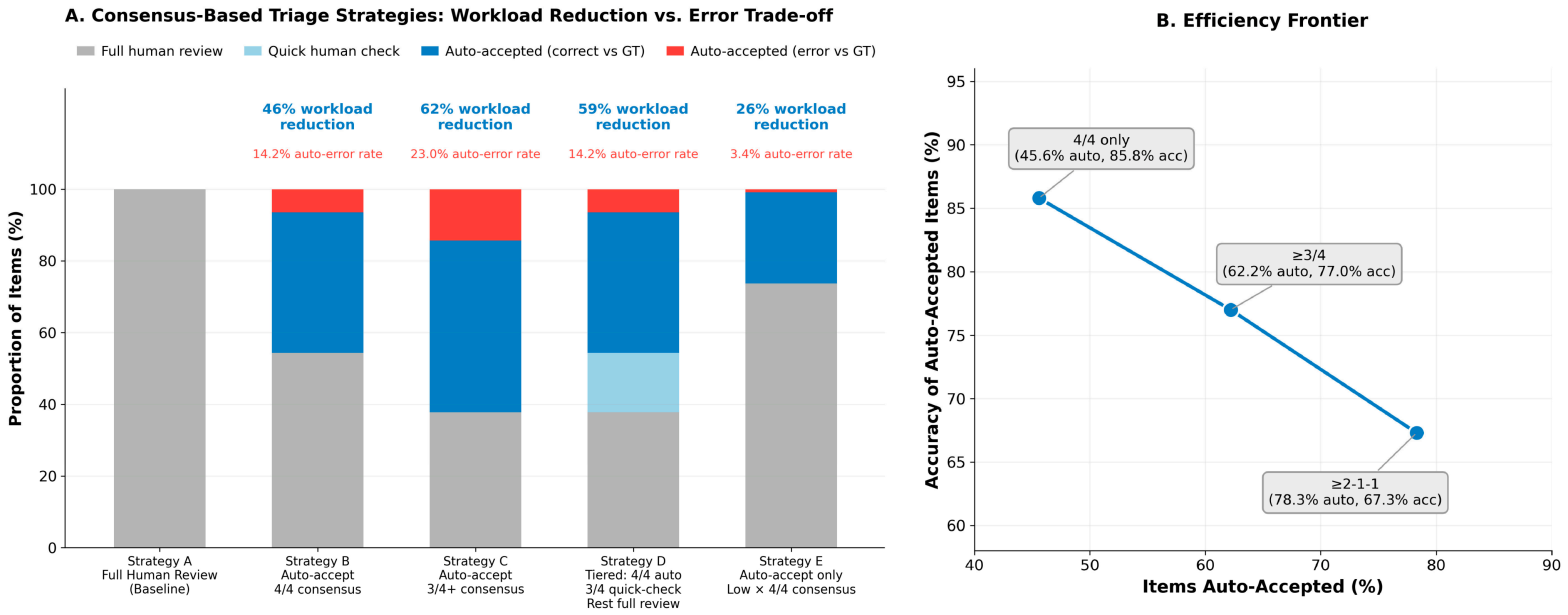
**Consensus-based triage strategies and the accuracy-automation trade-off.** (**A**) Item disposition under five strategies, from full manual review (A) to consensus-gated automation (B–E), annotated with effort-weighted workload reduction and the auto-acceptance error rate. The tiered strategy (D) achieved a 59% workload reduction while holding the auto-error rate at 14.2%. (**B**) Efficiency frontier across cumulative consensus thresholds (4/4 only, ≥3/4, ≥2-1-1): accuracy of auto-accepted items versus the proportion auto-accepted. Items lacking a plurality answer (2-2, 1/4) cannot be auto-accepted, so automation peaks at 78.3%.

These simulations trace an efficiency–accuracy frontier across the five strategies. The most conservative low-complexity strategy minimized the auto-accept error, whereas the tiered 4/4 auto-accept plus ¾ verification strategy maximized workload reduction while preserving review of intermediate-confidence items. The workflow-level distribution of the full review, quick check, and automatic acceptance across strategies is shown in Figure 6A, and the efficiency–accuracy frontier is shown in Figure 6B.

## 4. Discussion

This study evaluated whether LLM-assisted data extraction in neuroimaging AI can be embedded into a reliable workflow rather than assessed only as a model-level accuracy task. Three findings together support a complexity-stratified human–AI hybrid framework. First, exact-match evaluation substantially underestimates LLM performance: under prespecified semantic normalization, all four frontier models converged to a narrow 80.5–83.4% accuracy band, indicating that much of the apparent variation between models reflects surface-form differences rather than content-level extraction failure. Second, inter-model consensus operates as a meaningful but imperfect confidence signal: exact-match accuracy of the consensus answer increased monotonically from 29.6% at 2-1-1 plurality to 85.8% at 4/4 agreement, yet 14.2% of unanimously agreed items still disagreed with the expert reference. Third, unanimous agreement was highly reliable for low-complexity variables (96.6%), less reliable for medium-complexity variables (73.2%), and largely uninformative for high-complexity methodological variables (38.1%).

The convergence of all four models to nearly identical semantic-equivalence accuracy indicates that a substantial fraction of observed extraction failures reflect representational mismatches between model outputs and reference-standard encoding rather than true semantic failures. This pattern extends the representational mismatch effect previously documented for the main performance metric variable, where all four models scored 0% exact-match accuracy despite recovering the correct metric in approximately 90% of articles under semantic review [12]. These findings reinforce the need to complement exact-match scoring with prespecified semantic-equivalence review, particularly for variables that admit multiple valid lexical forms [2,13,14]. However, semantic normalization should be viewed as an evaluation safeguard, not as justification for automating variables that require methodological interpretation [5,6].

Inter-model consensus served as a useful but incomplete confidence signal. The monotonic relationship between consensus and ground-truth accuracy indicates that consensus carries genuine workflow value beyond single-model benchmarking [12]. Importantly, absence of consensus did not mean absence of the correct answer: the expert reference was semantically present among model outputs in 96.2% of 1/4 items and 92.6% of all no-consensus items. These cases are therefore best viewed as high-value review targets, where human adjudication selects from a candidate set that usually contains the correct value. This is consistent with prior evidence supporting redundancy and human adjudication rather than single-pass acceptance [2,6,15]. At the same time, even unanimous agreement was associated with a 14.2% exact-match mismatch rate relative to the reference standard, indicating that agreement alone cannot justify full automation. Comparable patterns of internally consistent but externally misaligned LLM performance have been reported in clinical decision-support settings, raising the possibility that consensus among LLMs may partly reflect correlated tendencies in output patterns rather than independent validation [16,17,18]. Thus, consensus should be treated as a triage signal rather than an independent surrogate for ground truth, especially for variables requiring interpretive judgment [5].

A central observation of this study is that the reliability of extraction is not determined by consensus level alone. Within the prespecified 7-8-7 framework, unanimous agreement was reliable for low-complexity variables, only moderately informative for medium-complexity variables, and largely uninformative for high-complexity methodological variables. This divergence indicates that consensus and variable complexity provide complementary, not interchangeable, information, consistent with prior extraction studies showing task-dependent reliability across clinical and evidence-synthesis contexts [13,19,20]. Consequently, automation rules should be stratified by complexity tier rather than governed by a single global consensus threshold. Specifically, this approach operationalizes a tiered workflow, in which low-complexity variables are designated for conservative auto-acceptance, medium-complexity variables are routed to targeted human verification, and high-complexity, inference-heavy variables remain human-led. This tiered model reduces routine extraction effort while reserving expert evaluation for interpretive and methodologically complex judgments, consistent with evidence that hybrid workflows outperform fully automated or fully manual approaches in structured clinical tasks [11,21,22].

The workflow simulations make the underlying efficiency–accuracy frontier explicit. The unanimous low-complexity subset represents the most conservative operating point, while the 4/4 auto-accept plus ¾ rapid-verification hybrid achieves the largest workload reduction while preserving human oversight of intermediate-confidence items. Thus, the appropriate operating point depends on whether the workflow prioritizes maximal error avoidance, maximal workload reduction, or a balance between the two. The complexity-dependent reliability observed here is consistent with broader healthcare assessments showing substantial heterogeneity across domains, tasks, and clinical settings [23,24]. These findings underscore the need for robust validation frameworks when implementing LLMs in clinical or research workflows [25,26].

The present findings reflect a fixed, non-optimized zero-shot workflow. Because the task involves repeated extraction of the same 22 variables, task-specific adaptation, supervised fine-tuning, alternative prompting, or different context construction could improve performance and alter the observed thresholds. Accordingly, the present findings should be interpreted as the zero-shot baseline performance of untuned general-purpose LLMs rather than as a general estimate of LLM capability or an upper bound on the performance of task-adapted extraction systems. Nevertheless, consensus and variable complexity remain useful calibration dimensions, although their thresholds should be re-estimated whenever the extraction configuration changes.

## 5. Limitations

Several limitations should be noted. First, the study evaluated a single zero-shot prompt without supervised fine-tuning, task-specific adaptation, alternative context construction, or prompt optimization; therefore, the results represent this configuration rather than the upper limit of LLM extraction performance. Second, the predefined 22-variable schema did not include a dedicated measure of demographic reporting or population representativeness. Because this study was a secondary analysis of an existing reference dataset and previously generated LLM outputs, adding such a variable would have required new manual annotation and model extraction. Future extensions should incorporate demographic-reporting variables. Third, the reference standard was constructed through multi-reviewer consensus and further curated for this benchmark. Although pre-consensus inter-rater agreement statistics, per-variable discrepancy proportions, and counts of third-reviewer adjudications were not systematically logged, the reference should be regarded as rigorous but not perfect. Fourth, hallucination cases, in which a model generates a plausible but factually unsupported value, were not systematically assessed because the reference standard did not encode “not reported” as a negative-control target. We thus suggest that future studies should include explicit negative-control variables to quantify hallucination rates in hybrid workflows. Fifth, the corpus comprised 91 neuroimaging AI articles drawn from the prior systematic review, and generalization to other specialties or review types remains to be established. Sixth, although no systematic preprocessing failures were identified in the born-digital PDFs used here, generalization of the pipeline to scanned, image-only, or low-quality source documents would require a separate document-processing robustness evaluation. Seventh, exact-match accuracy systematically underestimates true semantic performance on free-text and numeric variables admitting multiple valid representations, and accuracy figures should therefore be interpreted as conservative lower bounds, complemented by semantic-equivalence metrics. Workload reductions were based on predefined effort weights rather than measured reviewer time and should therefore be interpreted as indicative efficiency estimates. Finally, these findings reflect a snapshot of model capabilities as of 2 February 2026; given the pace of LLM evolution, periodic re-evaluation will be essential.

## 6. Conclusions

LLM-assisted extraction in the neuroimaging AI literature is most defensibly framed as a workflow design problem rather than a single-model accuracy task. Building on the prior systematic review and multi-model benchmark on the same corpus, the present study shows that a complexity-stratified human–AI hybrid framework, in which inter-model consensus and variable complexity are jointly used to guide triage, can substantially reduce routine extraction effort while preserving expert oversight of interpretively demanding variables. Low-complexity variables can be selectively automated under conservative consensus rules, medium-complexity variables benefit from LLM-assisted pre-extraction with targeted human verification, and high-complexity methodological variables continue to require expert supervision. The reported thresholds are configuration-specific and should be recalibrated when models or extraction strategies change.

## Figures and Tables

**Table 1:** Model-Level Impact of Semantic Normalization on Extraction Accuracy.

Model	*n*	Raw Rate	Semantic+	Δ (Absolute Increase %)	Absolute Gain (*n*)
Claude Opus 4.5	2002	51.3%	83.4%	+32.1%	643
GPT 5.2	2002	46.5%	80.5%	+34.0%	681
Gemini 3 Pro Preview	2002	56.4%	82.3%	+25.9%	518
Sonar Pro	2002	52.1%	80.5%	+28.4%	568

## Data Availability

The data presented in this study are available on request from the corresponding author.

## Supplementary Materials

The following supporting information can be downloaded at https://www.mdpi.com/article/10.3390/jcm15156005/s1. Supplementary Table S1: Classification of the 22 extraction variables by complexity tier, with rationale and extraction type; Supplementary Table S2: Sensitivity of estimated reviewer-workload reduction to the rapid-verification (quick-check) effort weight, hybrid triage strategy; Supplementary Table S3: Complete list of the 91 primary studies included in the LLM extraction corpus.

## Abbreviations

The following abbreviations are used in this manuscript:

AI	Artificial Intelligence
CLAIM	Checklist for Artificial Intelligence in Medical Imaging
CT	Computed tomography
JSON	JavaScript Object Notation
LLM	Large Language Model
MR	Magnetic resonance imaging
OCR	Optic Character Recognition
PDF	Portable Document Format
OCR	Optical Character Recognition
PROSPERO	International Prospective Register of Systematic Reviews
TRIPOD + AI	Transparent Reporting of a multivariable prediction model for Individual Prognosis or Diagnosis + Artificial Intelligence
