# Supplementary material for: How Often Do Large Language Models Agree with Each Other—And with the Truth? A Consensus- and Complexity-Stratified Analysis of Data Extraction for Neuroimaging AI
Source: J Clin Med. 2026 Aug 2;15(15):6005. doi: 10.3390/jcm15156005 (PMC13467224; doi:10.3390/jcm15156005)
Supplement: Supplementary file 1 [file jcm-15-06005-s001.zip › Supplementary File.pdf]

Supplementary Table S1. Classification of the 22 extraction variables by complexity tier, with rationale and extraction type.

Variable	Tier	Extraction type	Rationale
Publication year	**Low**	Direct categorical	Single stated value in a fixed location
AI type (DL/ML/hybrid)	**Low**	Fixed categorical	Small closed value set, explicitly stated
Claim of clinical applicability (yes/no)	**Low**	Binary	Explicit author statement
Performance metrics defined (yes/no)	**Low**	Binary	Presence/absence, directly checkable
Study design (retrospective/prospective/mixed)	**Low**	Fixed categorical	Usually explicitly stated
External dataset used (yes/no)	**Low**	Binary	Directly stated
Calibration metrics reported (yes/no)	**Low**	Binary	Presence/absence, directly checkable
Human comparator (yes/no)	**Medium**	Entity recognition	Requires locating comparison design; some judgment
Dataset type (single-center/multi-center/public)	**Medium**	Categorical + interpretation	Sometimes implicit; requires reading methods
Country of dataset origin	**Medium**	Entity extraction	May require inference from data source/affiliations
Data leakage risk (low/moderate/high/unclear)	**Medium**	Categorical + interpretation	Requires reading data-handling; partial judgment
Task type (classification/detection/segmentation/…)	**Medium**	Categorical	Multiple categories; occasional multi-task ambiguity
Imaging modality (MRI/CT/…)	**Medium**	Entity extraction	Usually stated but naming varies
Medical field/specialty	**Medium**	Categorical + interpretation	Sometimes inferred from clinical context
Journal	**Medium**	Entity extraction	Metadata, but free-text/canonical-form variability
Sample size	**High**	Numeric + disambiguation	Multiple cohort numbers; must identify primary cohort
CLAIM/TRIPOD+AI adherence (yes/partial/no/not reported)	**High**	Multi-domain judgment	Synthesis across several reporting domains
Split description clarity (yes/no)	**High**	Inference	Requires judging adequacy of split reporting
Ground-truth definition	**High**	Free-text synthesis	Multi-section, interpretive
Model architecture	**High**	Free-text synthesis	Free-text descriptor with wide lexical variation across reports
Validation type (internal/external)	**High**	Inference	Often implicit; requires inferring internal vs. external from the data design
Main performance metric	**High**	Free-text/numeric	Multiple reported metrics; requires identifying the primary one

Tiers were defined a priori. Low-complexity variables (n=7) are fixed categorical fields with a single canonical location; medium-complexity variables (n=8) require text extraction or entity recognition with minimal interpretation; high-complexity variables (n=7) require multi-section synthesis, clinical inference, or domain-level judgment.

Supplementary Table S2. Sensitivity of estimated reviewer-workload reduction to the rapid-verification (quick-check) effort weight, hybrid triage strategy.

Quick-check weight	Effective workload (% of baseline)	Workload reduction
0.10	39.5%	60.5%
**0.20 (used in main text)**	41.1%	**58.9% (~59%)**
0.30	42.8%	57.2%
0.50	46.1%	53.9%

Hybrid=4/4 auto-accept (weight 0) + 3/4 rapid verification (variable quick-check weight), full manual review of all remaining (2-1-1, 2-2, and 1/4) items (weight 1.00); n=1,994 consensus-evaluable items. Only the quick-check weight is varied; the auto-accepted set (910 items) and its error rate (14.2% exact-match; 4.7% after semantic normalization) are invariant across weights.
